# Fracture behavior of endodontically treated premolars restored with direct and indirect restorations: an in vitro study

**DOI:** 10.1186/s12903-026-08736-2

**Published:** 2026-06-13

**Authors:** Esraa Abdel Ghaffar El Gezawy, Samar Saeed Bedair, Khaled Farouk Abbas, Somaya Algizawi, Raghda Kamh

**Affiliations:** 1 Conservative and restorative dentistry department, Faculty of Dentistry, Galala University, Suez, Egypt; 2 Fixed Prosthodontics Department, Faculty of Oral and Dental Medicine, Egyptian Russian University, Cairo, Egypt; 3 Endodontics Department, Faculty of Oral and Dental Medicine, Egyptian Russian University, Cairo, Egypt; 4 Riyadh, Saudi Arabia; 5 Conservative and restorative dentistry department, Faculty of Oral and Dental Medicine, Egyptian Russian University, Cairo, Egypt

**Keywords:** Fracture resistance, Endodontically treated premolars, Short-fiber-reinforced resin-based composite, Nnanohybrid resin-based composite, CAD/CAM, Lithium Disilicate, Endocrown restoration

## Abstract

**Background:**

Endodontically Treated Teeth (ETT) have challenges in resisting fracture against occlusal forces. This study aimed to assess the fracture resistance (FR) of endodontically treated premolar teeth restored with an innovative self-adhesive bulk-fill resin-based composite (RBC) (Stela , SDI Limited, Australia), a short-fiber-reinforced composite (SFRC) (EverX flow, GC., Tokyo, Japan), a conventional nanohybrid (RBC) (Filtek Z250 XT, 3M ESPE, St. Paul, MN, USA) , and a Computer-Aided Design/Computer-Aided Manufacturing (CAD/CAM) Lithium Disilicate (Emax, Dentsply Sirona, Bensheim, Germany) Endocrown.

**Methods:**

For the FR test, 44 human extracted maxillary premolars were included in the study. Endodontic treatment was done, and a single operator prepared standardized mesio‑occlusal‑distal (MOD) cavities in the teeth and divided into four groups (n=11) according to the restorative material used. Group (F1): ETT restored with Stela resin-based composite, which was inserted into the cavity as a single 5 mm increment. Group (F2): ETT restored with (EverX Flow) composite,which was packed and light-cured for 20 seconds, leaving a 2 mm occlusal layer to be restored with nanohybrid composite (Filtek Z250 XT). Group (F3): ETT restored with Filtek Z250 XT resin-based composite only. Group (F4): ETT restored with (CAD/CAM Cerec MCXL) Lithium Disilicate endocrown, preparations were scanned with the Omnicam, and restorations were designed using CEREC Premium Software (version 4.4). For direct restoration groups the proximal walls were reconstructed by using a tofflemire matrix retainer and band. All restored teeth were subjected to FR testing using a universal testing machine ( Bluehill Lite Software on an Instron® / Each specimen’s failure load was recorded in Newton). Each specimen was individually mounted on the computer-controlled machine equipped with a 5 kN load cell, with the acrylic block secured to the lower fixed head.

**Results:**

Statistical analyses were conducted using SPSS 27®, GraphPad Prism®, and Microsoft Excel 2016. Data normality was assessed with the Shapiro-Wilk and Kolmogorov-Smirnov tests, which indicated a nonparametric distribution. Comparison between groups revealed a significant difference (P<0.0001), with Group F4 (Endocrown) demonstrating the highest performance (1427.73 ± 156.96), compared to all direct restoration materials. In contrast, there were no significant differences among the three direct restoration groups despite slight differences in their means. Group F2 (EverX+Z250, mean: 619.87 ± 186.41) was higher than Group F1 (Stella, mean: 561.75 ± 173.74) with *P* = 1.000, and higher than Group F3 (Z250, mean: 452.22 ± 202.72) with P = 0.471. Group F1 (Stella) exceeded Group F3 (Z250), but this difference was not statistically significant (*P* = 0.838).

**Conclusions:**

While lithium disilicate endocrowns exhibit superior fracture resistance, direct restorative materials may serve as a clinically acceptable alternative for routine applications. Nevertheless, these findings should be interpreted with caution, given the inherent limitations of the in vitro study design.

## Background

Endodontically Treated Teeth (ETT) generally have lower resilience and Fracture Resistance (FR). This may be due to loss of coronal tooth structure and vitality after caries and endodontic procedures. The type of restorative technique significantly affects tooth FR. Traditionally, restoring ETT involves a full-coverage crown or a post-and-core crown. However, preparing teeth for a crown removes significant enamel and dentin, compromising sound tooth structure. This is a major drawback [1]. Extensive mesio-occlusal-distal (MOD) cavity preparations, lower tooth toughness by up to 54% compared to sound teeth. These complex cavities are prone to harmful occlusal forces and microcracks [2]. Maxillary premolars are more likely to fracture vertically than molars because of higher masticatory forces. Thus, endodontically treated maxillary premolars with complex cavities should be restored promptly to maintain tooth integrity, aesthetics and function [3].

Against this backdrop, it is important to note that stress transfer differs between intact and restored teeth. Accordingly, multiple restorative strategies have been recommended for endodontically treated teeth with MOD cavities. These strategies encompass a wide range of restorative options, each presenting distinct clinical indications, benefits, limitations, and technical considerations [4].

Within this framework, a resin-based composite (RBC) is often chosen to repair teeth treated with root canal therapy (RCT). Its flexibility is similar to that of the tooth’s inner layer (dentin), so it can bend under pressure and withstand stress. But when the material hardens, it can shrink, which is still a problem. If this shrinkage creates more force than the glue holding the material to the tooth, the bond can fail, and the tooth is not strengthened. If the bond is very strong, the tooth itself could crack under stress [5].

Adding fibers to the composite matrix creates an innovative fiber-reinforced composite. Fibers used can be glass, carbon, or polyethylene. EverX Posterior arose from research on dentin substitute. It is a biomimetic material composed of millimeter-scale E and barium glass fibers, arbitrarily distributed within the resin matrix. Its poor aesthetics and difficult workability led to EverX Flow. EverX Flow, a low-viscosity alternative, uses dentine color and is easier to handle. This is because its E-glass fibers are micrometer-sized, and the content increased to 25% by weight. The result is better fracture toughness [2].

To minimize these effects, each increment should be meticulously applied to ensure optimal adaptation and adhesion, particularly in deep and extensive cavities. However, this method also has drawbacks: it may cause internal flexure, risk contamination of intermediate layers, or trap voids between them [6]. In response to these challenges, “bulk-fill” materials were recently introduced. Utilizing better photo-initiators and higher translucency, they enable deeper curing. As a result, bulk-fill composites may be applied in increments of 4–5 mm and polymerized in a single curing cycle, thereby reducing shrinkage stress [5]. Further advancing this field, a self-adhesive bulk-fill Stela composite was recently developed to address polymerization stresses in conventional RBCs. Its goal is to improve the bond between restoration and tooth structure [7].

Endocrowns represent a reliable alternative to conventional full-coverage crowns for the restoration of endodontically treated molars. Many authors have shown this [8]. It consists of a monolithic ceramic restoration featuring a butt-joint margin and an internal retention cavity within the pulp chamber, resulting in a unified crown–core structure. Pulp chamber walls provides retention by adhesive bonding. This preserves more teeth by following a decay-oriented design [9]. Recently, Endocrown has also been considered for premolars. However, its biomechanical performance and durability are still debated [10].

Recent ceramic advances and CAD/CAM technologies meet high aesthetic demands. They also address issues found in traditional materials, such as low tensile strength or brittleness [11]. Lithium Disilicate is now widely used due to its adhesive properties [12]. It is minimally invasive, aesthetic, and highly effective [13]. Literature discusses various occlusal preparation designs and depths [14]. Some studies found that anatomic cusp-reduction designs strengthen endodontically treated maxillary premolars the most. There is currently no agreement about the best restorative material or preparation design for ETT [15]. This study compared the FR of four restorations: innovative Stela, a self-adhesive bulk-fill composite; EverX flow, an SFRC; Filtek Z250 XT, a nano-hybrid resin composite; and a CAD/CAM Lithium Disilicate endocrown. The null hypothesis proposed that there would be no significant differences in fracture resistance among the four restorative types when applied to endodontically treated maxillary premolars.

## Materials and methods

The used materials are listed in Table 1.

**Table 1:** Details of the selected dental materials, including composition and manufacturer

Selected materials	Composition	Manufacturer
STELA (SDI STELA Automix, self-adhesive resin-based bulk-fill material)	**Catalyst**: Contains barium glass, silica, ytterbium trifluoride (YbF₃), urethane dimethacrylate, along with polymerization initiators and stabilizing agents. **Base**: Composed of strontium fluoroaluminosilicate glass, agglomerated ytterbium trifluoride (YbF₃), silica, calcium aluminate (Al₂CaO), urethane dimethacrylate, in addition to initiators and stabilizers.	SDI Limited, Victoria, Australia
STELA Primer	Contains 10-MDP, dimethacrylate, methyl ethyl ketone (MEK), water, along with polymerization initiators and stabilizers.	SDI Limited, Victoria, Australia
Filtek Z250 XT nanohybrid composite	**Resin matrix**: Composed of Bisphenol A glycol dimethacrylate (Bis-GMA), Urethane dimethacrylate (UDMA), Ethoxylated Bisphenol A glycol dimethacrylate (BIS-EMA), Polyethylene glycol dimethacrylate (PEGDMA), and Triethylene glycol dimethacrylate (TEGDMA). **Fillers**: Make up 82% by weight and include surface-treated zirconia/silica, as well as 20-nanometer non-agglomerated and non-aggregated surface-modified silica particles.	3 M ESPE, St. Paul, MN, USA
3 M™ Scotchbond™ Etchant	37% phosphoric acid	3 M ESPE, St. Paul, MN, USA
Adper single bond 2	Contains ethanol, water, Bisphenol A glycol dimethacrylate (Bis-GMA),5nmsilane-treatedcolloidalsilica,2-hydroxyethylmethacrylate(HEMA), glycerol1,3dimethacrylate, a methacrylate-functional copolymer of polyacrylic and polyitaconic acids, and diurethane dimethacrylates.	3 M ESPE, St. Paul, MN, USA
EverX Flow	resin matrix: 26 wt% composed of UDMA, bis-GMA, and TEGDMA. Fillers: barium borosilicate 68wt, 0.7 μm in size.E-glass micrometer-scale short fibers total 25wt% with average size 0.2–0.3 mm	GC Corp., Tokyo, Japan
E-max CAD	Lithium Disilicate glass ceramic Emax CAD A2 HT, finished, polished, and crystallized as recommended by the manufacturer.	Ivoclar Vivadent, Liechtenstein
G-CEM	Dual-cure self-adhesive universal resin luting cement capsules	GC Japan

### Research ethical approval

This in vitro study received approval from the Research Ethics Committee of the Faculty of Dentistry, Ain Shams University (FDASU-REC), under the ethical approval number FDASU-RecER102505 (November 16, 2025).

### Study design

**Fig. 1:**
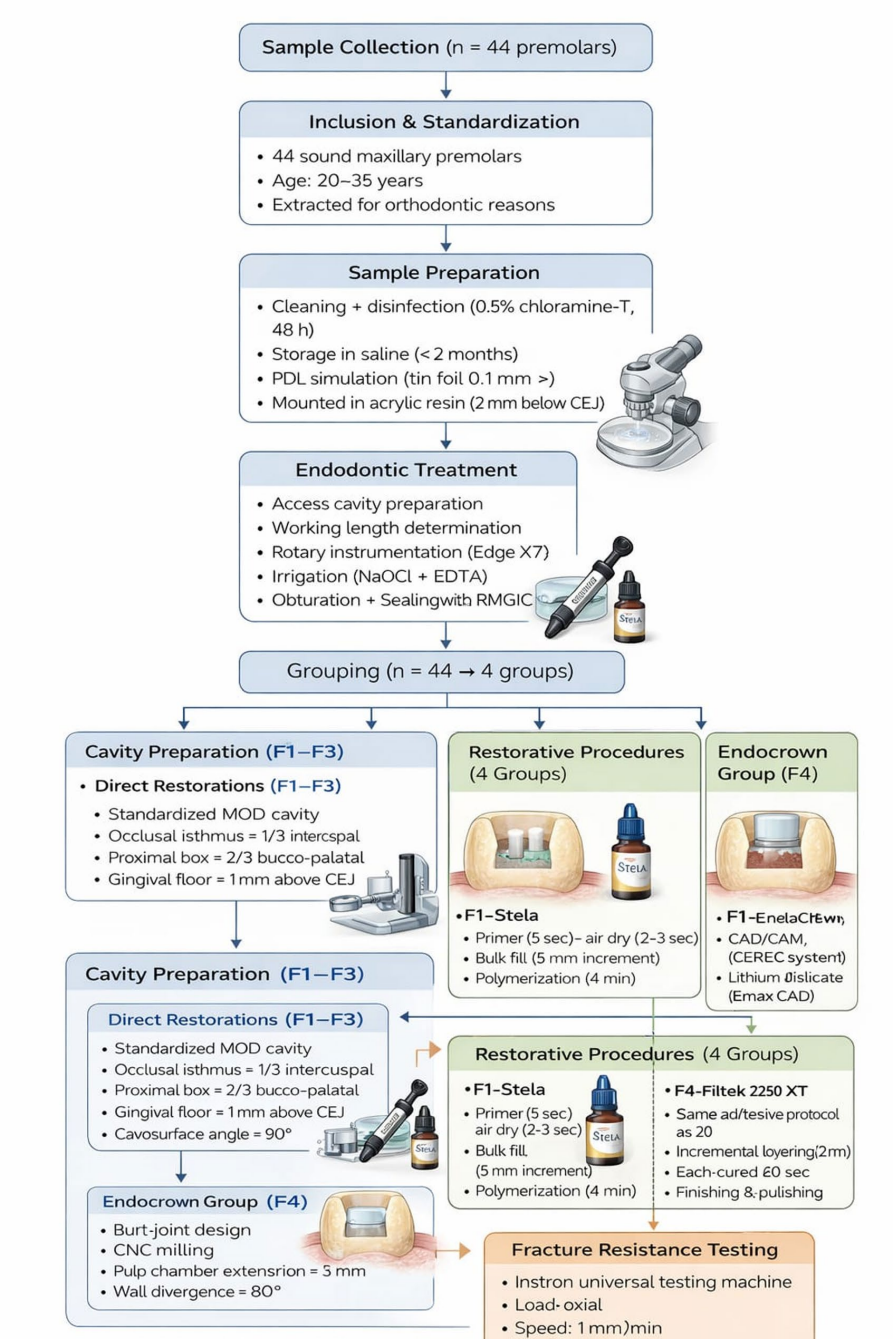
A flowchart illustrating the sequential steps of the methodology

Forty-four sound maxillary permanent premolars were included in the study. Teeth were collected from patients undergoing orthodontic treatment, aged 20–35 years. Endodontic treatment was carried out, followed by the preparation of standardized MOD cavities in the teeth. For the Endocrown group, teeth were prepared using a horizontal butt-joint design and restored with Endocrowns of 2 mm thickness. Samples were categorized into four main groups (*n* = 11) according to the restorative material used. Group (F1): ETT restored with Stela RBC. Group (F2): ETT restored with EverX Flow composite and Filtek Z250 XT RBC. Group (F3): ETT restored with Filtek Z250 XT RBC only. Group (F4): ETT restored with (CAD/CAM Cerec MCXL) Lithium Disilicate Endocrown. Fracture resistance of all restored teeth was evaluated using a universal testing machine. Figure 1.

### Sample size calculation

The sample size was determined using data from a previous study that assessed the fracture resistance of endodontically treated premolars restored with various restorative materials. (Eapen et al., 2017) [16]. According to their results, normally distributed data were obtained, and a minimum of 10 specimens per group was sufficient to assess for a statistically significant difference at the 95% confidence level (α = 0.05) and 80% power (β = 0.20) (Hafez et al., 2025) [17]. Considering the present study design, which includes four groups (novel self-adhesive bulk-fill composite, SFRC, conventional nano-hybrid RBC, and Lithium Disilicate Endocrown), the required total sample size was calculated as follows: *n* = 10 specimens per group × 4 groups = 40 specimens. To account for possible specimen loss during preparation or testing, the sample size was increased by 10%, yielding a total of 44 specimens. (11 per group).

### Sample preparation

This study utilized 44 intact, freshly extracted human maxillary premolars from individuals aged 20–35 years. The teeth were atraumatically extracted for orthodontic reasons from healthy patients at the Department of Oral Surgery, Faculty of Oral and Dental Medicine, Egyptian-Russian University. All teeth were of similar dimensions, measured mesiodistally and buccolingually using a digital caliper, and only teeth with complete root formation were included. Teeth were inspected for caries, existing restorations, fractures, or cracks. Following the removal of soft-tissue remnants with a hand scaler, the selected teeth were disinfected in 0.5% chloramine-T solution for 48 h, then polished using a rubber cup with a fine pumice-water slurry [6]. Then they were stored in normal saline at room temperature for up to 2 months after extraction. Preservatives were not used, as they could modify the dentin protein composition and potentially affect the bonding process [18]. A 0.1 mm layer of tin foil was placed around the roots, extending 1 mm below the cementoenamel junction (CEJ), to simulate the periodontal ligament [19]. Teeth were mounted in an auto-polymerized acrylic resin block. Teeth were positioned 2 mm below the CEJ to mimic the alveolar bone level, with their long axes aligned vertically to the mold walls and their occlusal surfaces parallel to the plane of the acrylic resin mold [18].

### Endodontic treatment

A standardized conventional access cavity was prepared in the occlusal surface of premolars using the access cavity set (round bur size 2 for initial penetration and pulp exposure, and a round-ended tapered stone to complete deroofing and flaring). Canals were scouted with a K-type Stainless steel file size 10 to check their patency. Coronal flaring was made by using the orifice opener taper 8% NiTi rotary files Edge X7. Working lengths were recorded using the radiographic technique. The glide path was created using K-type files sizes 10 and 15, and irrigation of the root canal system was performed with 10 ml of 2.25% sodium hypochlorite solution. Mechanical preparation was performed using Edge X7 heat-treated NiTi rotary files with a 4% taper in the crown-down direction. Apical preparation was enlarged to sizes 30, 35, and 40, taper 4%, according to the initial canal diameter. Continuous irrigation with 2.25% sodium hypochlorite was maintained throughout the cleaning process. A final flush was performed using 17% EDTA, the canals were subsequently dried using paper points matched to the master cone size of each root canal Root canal obturation was performed using the lateral compaction technique with AD-Seal epoxy resin sealer. Excess gutta-percha and sealer were removed up to the canal orifices, and the cavity walls were cleaned with alcohol-moistened cotton. A thin layer of resin-modified glass ionomer cement (GC, Tokyo, Japan) was then applied to seal the cavity.

### Teeth grouping

In this in vitro study, 44 endodontically treated maxillary premolars were assigned to four groups according to the restorative material used (*n* = 11 per group). Thirty-three teeth were prepared with MOD cavities for direct restorations, while 11 teeth were prepared for Lithium Disilicate (Emax) Endocrowns.

### Cavity preparation for endodontically treated teeth receiving direct restorations

**Fig. 2:**
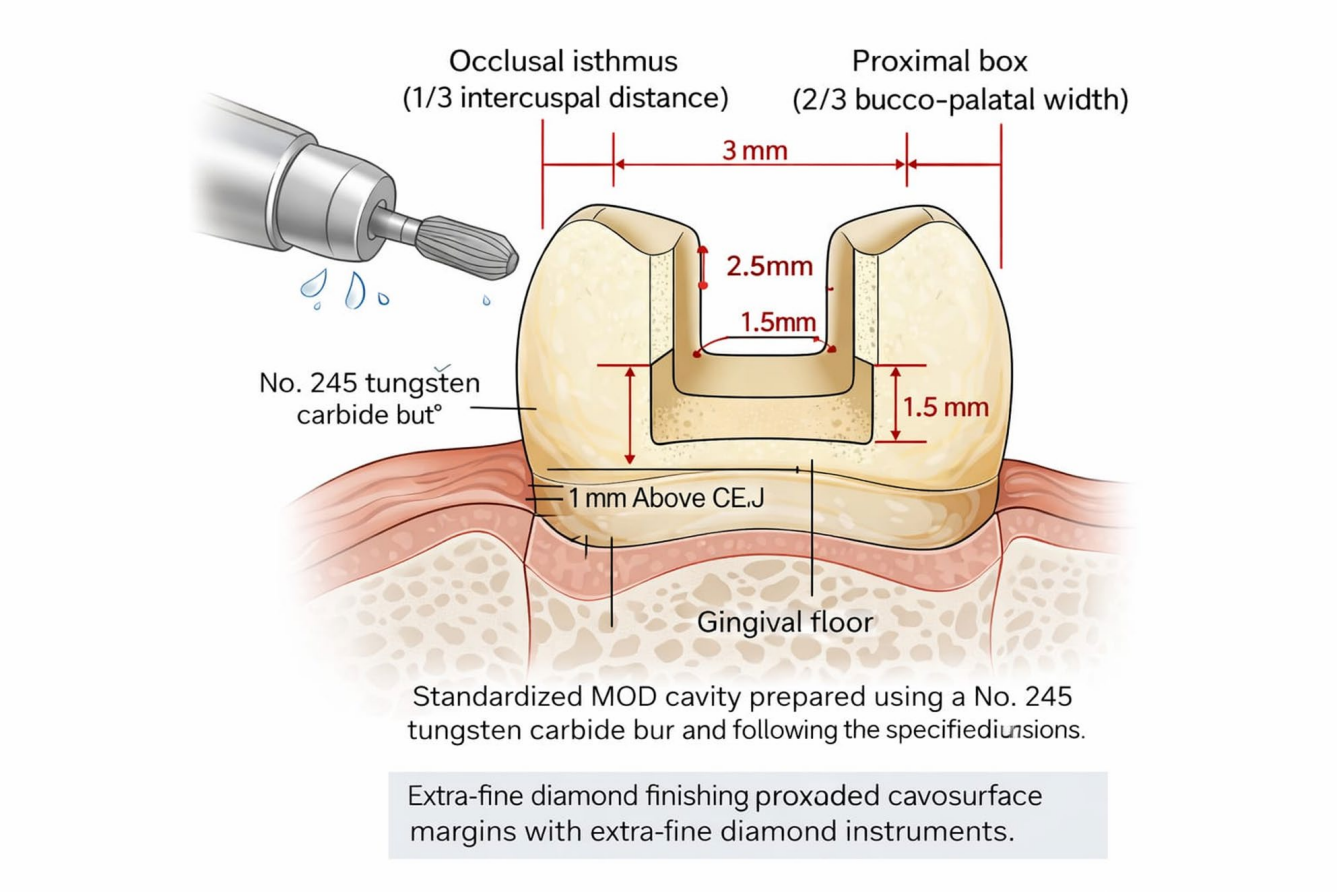
Illustration of cavity preparation for endodontically treated teeth receiving direct restorations

A single operator prepared standardized MOD cavities using a No. 245 tungsten carbide bur in a high-speed handpiece (Dentsply Sirona GmbH, Konstanz, Germany) with abundant air-water cooling. Burs were replaced after every four preparations to maintain cutting efficiency. The occlusal isthmus measured one-third of the intercuspal distance, while the proximal box extended two-thirds of the bucco-palatal width. The gingival floor was positioned 1 mm above the cementoenamel junction (CEJ), and cavosurface margins were prepared at a 90° angle. Finishing was performed with extra-fine diamond instruments to create rounded line and point angles. All preparation dimensions were verified using a periodontal probe. Figure 2.

### Cavity preparation for endodontically treated teeth restored with emax (CAD/CAM) lithium disilicate endocrowns

Endodontically treated teeth were prepared using a butt-joint design with a Computerized Numerical Control (CNC) milling machine (C.N.C Premium 4820, imesicore, Eiterfeld, Germany) to ensure standardized dimensions. The CNC machine was set to reduce the occlusal surface, creating a pulp chamber with a retention cavity extending 3 mm from the central groove and walls diverging at an 80° angle.

### Restorative procedures

**Fig. 3:**
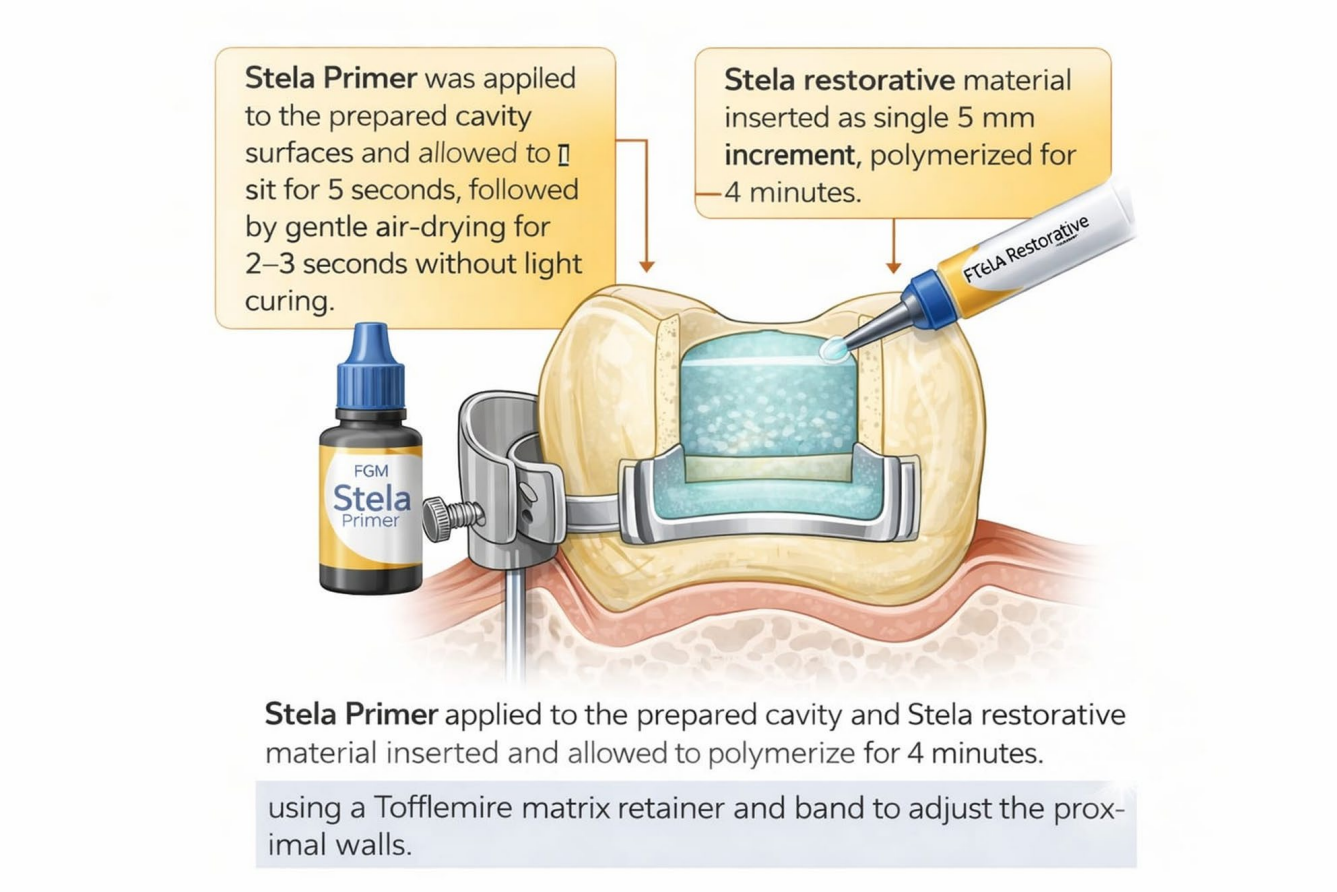
Illustration of ETT teeth were restored with Stela

- ◦ *Group (F1)* ETT teeth were restored with Stela (self-adhesive bulk-fill RBC) as per the manufacturer’s guidelines.
  - ◦ Stela Primer was applied to the prepared cavity surfaces and allowed to sit for 5 s, followed by gentle air-drying for 2–3 s without light curing.
  - ◦ Stela restorative material was inserted into the cavity as a single 5 mm increment, ensuring proper contact with the primer at the margins, and allowed to polymerize for 4 min. A Tofflemire matrix retainer and band were used to adjust the proximal walls. Figure 3

- ◦ *Group (F2)* ETT were restored with Ever X Flow + Filtek Z250 XT as per the manufacturer’s guidelines [3].

**Fig. 4:**
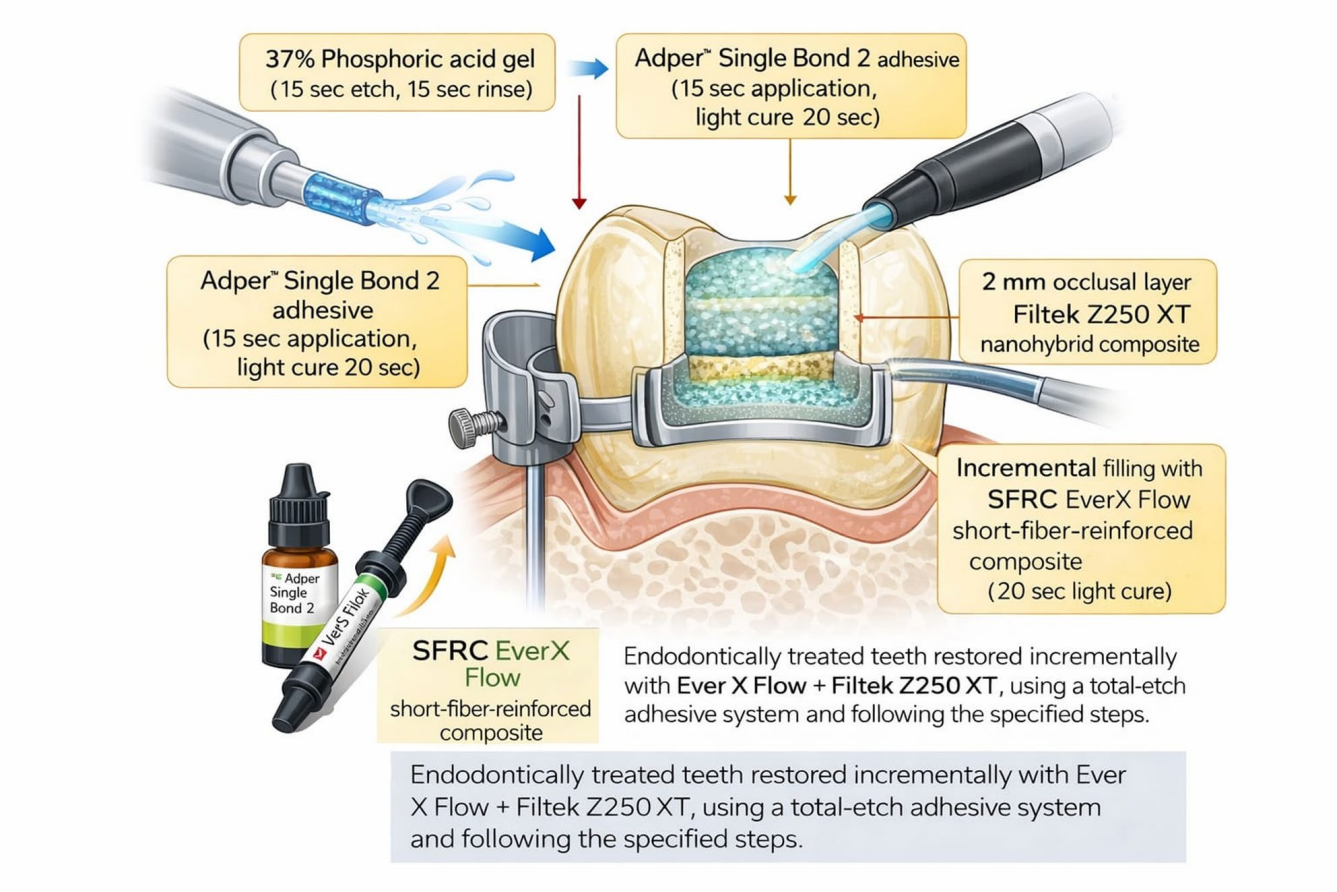
Illustration of ETT teeth were restored with Ever X Flow + Filtek Z250 XT

A total-etch adhesive system was employed. A 37% phosphoric acid gel was applied for 15 s and then rinsed for an additional 15 s. The cavity was gently air-dried with an oil-free air blast. Adper Single Bond 2 was applied with a microbrush using gentle agitation for 15 s, followed by light air-thinning for 5 s and light curing for 20 s. The proximal walls were reconstructed using a 1.5 mm layer of nanohybrid resin-based composite (Filtek Z250 XT) with the aid of a Tofflemire matrix retainer and band. The cavity was then converted to a Class I configuration and incrementally filled with short-fiber-reinforced composite (SFRC, EverX Flow), which was packed and light-cured for 20 s, leaving a 2 mm occlusal layer to be restored with nanohybrid composite (Filtek Z250 XT). Figure 4*.*

- *Group (F3)* ETT were restored with Filtek Z250 XT (nanohybrid RBC restoration) as per the manufacturer’s guidelines.

The adhesive protocol followed that of group F2. The proximal boxes were restored first using one horizontal and two oblique increments, each 2 mm thick as measured with a periodontal probe. Each increment was light-cured for 40 s. After removing the matrix band, additional curing was performed on the buccal and lingual surfaces to ensure complete polymerization and better simulate clinical conditions. Light curing was carried out using an LED unit (Elipar S10, 3 M Oral Care) with a wavelength range of 430–480 nm and an intensity of 1200 mW/cm². The restorations were then finished immediately under water cooling with diamond finishing burs (size 2, Apple Dental, China) and were polished using fine-grit Sof-Lex discs (KerrHawe SA, 6394 Bioggio/Switzerland).

- *Group F4*: Endodontically treated teeth were restored with Emax Endocrowns fabricated using the CEREC AC system (Dentsply Sirona, Bensheim, Germany). The preparations were scanned with the Omnicam, and restorations were designed using CEREC Premium Software (version 4.4). Standardized Endocrowns were milled using the CEREC MCXL milling machine. To maintain consistent form and anatomy, the restorations were positioned using only the software’s "position" tools (translation and rotation), without altering the original design generated by the software.

### Endocrown fabrication

The restorations were subsequently milled from Lithium Disilicate glass ceramic Emax CAD A2 LT (Ivoclar Vivadent, Liechtenstein), finished, polished, and crystallized according to the manufacturer’s instructions. Occlusal thicknesses were then verified using a digital caliper.

### Endocrown cementation

Endocrowns were cleaned in an ultrasonic bath with 99% isopropanol for 3 min, and the prepared teeth were polished with fluoride-free pumice for 15 s, then thoroughly rinsed with water for another 15 s. The bonding surfaces of the Endocrowns were etched with 9.5% hydrofluoric acid gel (Porcelain Etch, Ultradent Products, South Jordan, UT, USA) for 20 s, followed by thorough rinsing for 20 s and drying with oil-free compressed air. The surfaces were then treated with a silane primer (Porcelain Silane, Ultradent Products) and allowed to react for 60 s. The enamel of all preparations was selectively etched with 37% phosphoric acid for 30 s, rinsed, and dried. Self-adhesive resin cement (G-Cem, GC, Japan) was applied to the intaglio surfaces of the Endocrowns, which were seated on the corresponding preparations using static finger pressure, followed by axial loading with a 1 kg weight using a custom-designed device. The Endocrowns were kept under static load for 5 min, followed by an initial light curing (Elipar, 3 M ESPE) for 2 s. Excess cement was then removed with a scaler, and final light curing was performed for 20 s on each surface.

### Fracture resistance testing

**Fig. 5:**
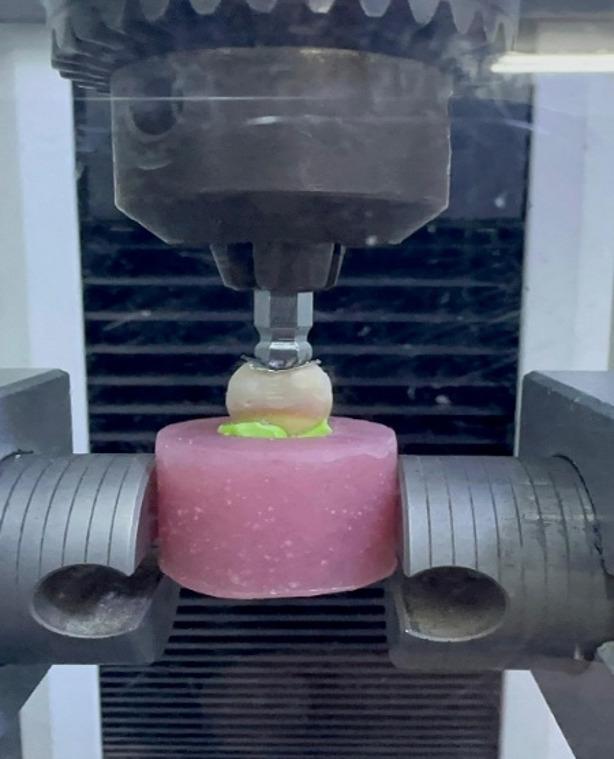
The mounted endodontically treated premolar in an acrylic resin block and adjusted for the Fracture resistance test

Fracture testing was conducted using Bluehill Lite Software on an Instron® universal testing machine (Model 3345; Instron Industrial Products, Norwood, MA, USA). Each specimen was individually mounted on the computer-controlled machine equipped with a 5 kN load cell, with the acrylic block secured to the lower fixed head (Fig. 5). Crowns were loaded axially using a 2 mm-diameter stainless-steel ball attached to the upper movable head, applying a continuous static compressive force at a crosshead speed of 1.0 mm/min until failure. Each specimen’s failure load was recorded in Newton.

### Statistical analysis

Statistical analyses were conducted using SPSS 27^®^, GraphPad Prism^®^, and Microsoft Excel 2016. Data normality was assessed with the Shapiro-Wilk and Kolmogorov-Smirnov tests, which indicated a nonparametric distribution. Consequently, group comparisons were performed using the Kruskal-Wallis test, followed by Dunn’s post hoc test for pairwise comparisons. The significance threshold was set at *P* ≤ 0.05.

## Results

### Evaluation of fracture resistance (Newton)

**Fig. 6:**
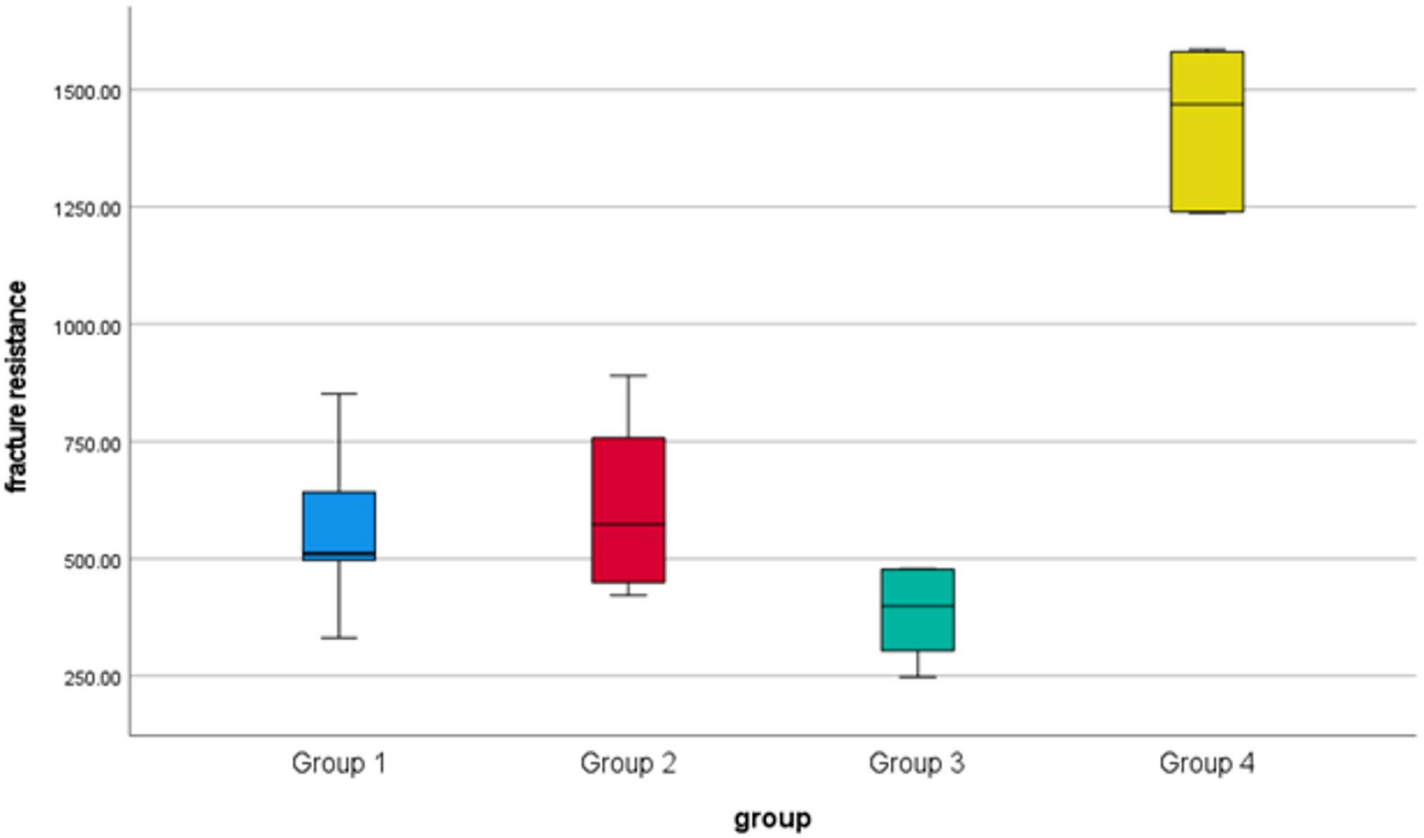
Box plot represents descriptive statistics of fracture resistance

**Fig. 7:**
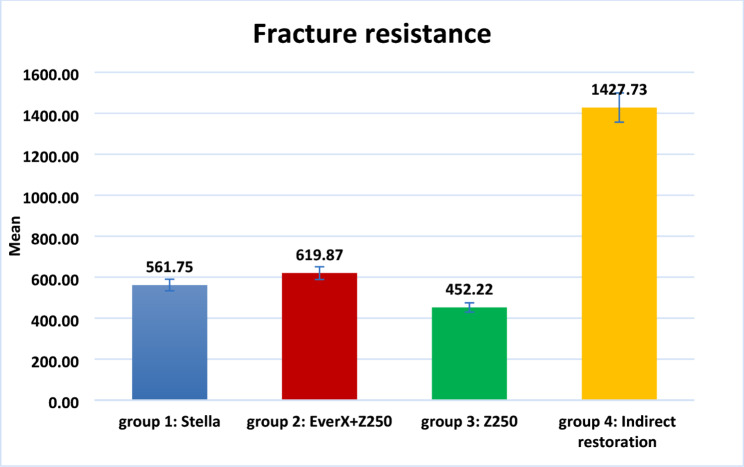
A Bar chart represents the comparison between groups regarding fracture resistance

Descriptive results of fracture resistance in all groups were presented in Table 2 and Fig. 6. Comparison between groups revealed that there was a significant difference between them (P<0.0001), followed by pairwise comparisons in Table 3 and Fig. 7, which showed that Group F4 (Indirect Restoration) demonstrated the highest performance, with a mean of (1427.73 ± 156.96), significantly exceeding all direct restoration materials by 808 to 976 units (P = 0.002 to 0.0001). In contrast, there were no significant differences among the three direct restoration groups despite slight differences in their means. Group F2 (EverX+Z250, mean: (619.87 ± 186.41)) performed 58 units higher than Group F1 (Stella, mean: (561.75 ± 173.74)) with P = 1.000, and 168 units higher than Group F3 (Z250, mean: (452.22 ± 202.72)) with P = 0.471. Similarly, Group 1 (Stella) exceeded Group 3 (Z250) by 110 units, but this difference was not statistically significant (*P* = 0.838).

**Table 2:** Descriptive statistics and overall comparison of fracture resistance (Newton) using the Kruskal-Wallis test

	Minimum	Maximum	Median	Mean (Newton)	Standard Deviation	*P* value
Group F1: Stella	331.02	851.38	511.08	561.75 ^a^	173.74	< 0.0001*
GroupF2: EverX+Z250	422.21	890.30	573.76	619.87 ^a^	186.41	
Group F3: Z250	247.56	825.41	399.13	452.22 ^a^	202.72	
GroupF4: Indirect restoration	1237.37	1586.49	1469.17	1427.73 ^b^	156.96	

Means with different superscript letters were significantly different (*P *≤ 0.05)

*Significant difference as* P*≤0.05

**Table 3:** Pairwise comparisons using Dunn’s test

Pairwise Comparisons of Groups	Test Statistic	Std. Error	Std. Test Statistic	*P* value
Group F1 vs. Group F2	-1.545	5.477	-0.282	1.000
Group F1 vs. Group F3	8.091	5.477	1.477	0.838
Group F1 vs. Group F4	-19.818	5.477	-3.618	0.002*
Group F2 vs. Group F3	9.636	5.477	1.759	0.471
Group F2 vs. Group F4	-18.273	5.477	-3.336	0.005*
Group F3 vs. Group F4	-27.909	5.477	-5.095	0.0001*

^*^Significant difference as *P*≤0.05

## Discussion

Restoring endodontically treated teeth (ETT) continues to be a clinical challenge. The structural integrity of these teeth is often compromised by extensive decay, traumatic injury, or existing restorations, further weakened by the removal of the pulp chamber roof during access cavity preparation.

This study evaluated the fracture loads of ETT when restored using four different materials and techniques:

- *Stela*: A novel, chemically self-adhesive bulk-fill resin-based composite (RBC).
- *EverX Flow*: A short-fiber reinforced composite (SFRC).
- *Filtek Z250 XT*: A traditional nanohybrid RBC used as a control.
- *CAD/CAM Lithium Disilicate*: Applied as an indirect endocrown restoration.

The results demonstrated significant variations in fracture resistance across the tested groups; consequently, the null hypothesis which predicted no difference between the materials was rejected.

Research indicates that chemically-cured, self-adhesive bulk-fill composites outperform traditional materials in large Mesio-Occlusal-Distal (MOD) restorations, particularly regarding fracture resistance and the management of polymerization shrinkage stress [5]. The flowable consistency of these materials facilitates superior internal adaptation to the cavity’s floor and walls, which in turn enhances marginal sealing.Furthermore, longitudinal clinical data over three- to five-year periods suggest that the bulk-fill method provides clinical outcomes comparable to the traditional incremental layering technique [6].

A critical concern with standard composite restorations in deep cavities is the development of cracks that can lead to catastrophic, non-restorable fractures. However, utilizing a “bilayer” approach incorporating short-fiber reinforced composites (SFRCs) has been shown to not only increase the tooth’s overall resistance to breaking but also to promote “repairable” fracture patterns if failure does occur [12].

An endocrown serves as a monolithic, adhesive restoration that gains its stability by anchoring directly into the internal anatomy of the pulp chamber. This design utilizes the internal walls of the chamber for micromechanical retention, eliminating the need for more invasive preparation. Modern endocrowns are frequently produced through digital workflows, specifically utilizing computer-aided design and manufacturing (CAD-CAM) technologies.

This restorative approach has gained significant clinical popularity due to several distinct advantages: Conservative Preparation: It prioritizes the maximum preservation of remaining natural tooth structure. Simplified Biomechanics: There is a reduced requirement for auxiliary retentive features like posts or pins. Clinical Efficiency: The streamlined workflow requires fewer clinical steps, saving both chairside time and patient costs.

Lithium disilicate is considered an ideal substrate for the construction of endocrowns. This is largely attributed to its excellent aesthetic characteristics, which closely mimic the translucency and shade of natural dentition. Furthermore, its robust flexural strength provides the necessary mechanical durability to withstand occlusal forces in the posterior region [20].

The FR of endodontically treated maxillary premolars restored with one indirect Endocrown restoration and three direct restorative materials was assessed in this study. The indirect Endocrown restoration significantly surpassed all direct composite restorations, according to the data, which showed a statistically significant difference in fracture resistance among the four restoration groups (*P* < 0.0001). Despite slight differences in their mean fracture resistance values, no statistically significant differences were found among the three direct restoration groups (F1, F2, and F3), in contrast to the apparent advantage of the Endocrown restoration.

Group F4 showed the highest fracture resistance, significantly exceeding all direct restorative protocols. This finding is strongly supported by Shams et al. (2025), who reported exceptionally high fracture loads for Lithium Silicate–based Endocrowns, ranging from 1360 N to over 1850 N, depending on the ceramic type, very similar to the 1427 N mean recorded in the current study. Their work emphasized that Lithium Disilicate and advanced Lithium Silicate ceramics consistently outperform other restorative materials due to their high flexural strength, crack-deflection behavior, and superior adhesive bonding potential [21]. Kazemi-Yazdi et al. (2023) further demonstrated that Lithium Disilicate Endocrowns can achieve fracture resistance values comparable to or even higher than sound teeth, particularly when preparation design optimizes ferrule support [20].

Group F2 showed the highest FR value among the direct groups (619.87 ± 186.41 N), followed by Group F1 and then Group F3. The superior performance observed in Group F2 is likely due to the unique properties and high fiber concentration in the flowable short-fiber-reinforced composite (SFRC). Several critical variables dictate the efficacy of fiber reinforcement, such as:

- *Composition and Interface*: The specific resin chemistry, the quality of fiber impregnation, and the strength of the bond between the fibers and the polymer matrix.
- *Physical Distribution*: The total weight of the fibers, as well as their specific placement and orientation within the restoration.
- *Geometric Factors*: The “aspect ratio,” defined as the ratio of a fiber’s length to its diameter (l/d).

In high-performance reinforced materials, the aspect ratio is a vital metric, as it directly influences flexural modulus, tensile strength, and overall reinforcing efficiency.

A novel short-fiber reinforced composite (SFRC) has been engineered specifically for use in high-stress clinical scenarios. This material incorporates millimeter-scale E-glass fibers in a random orientation, embedded within a unique semi-interpenetrating polymer network (semi-IPN). This specific matrix architecture ensures robust internal adhesion and acts as a structural barrier that inhibits both the initiation and the spread of cracks. Consequently, these features significantly bolster the material’s overall mechanical durability and performance [3, 22]. Several analyses of fiber-reinforced composites confirm our conclusion. Although the testing has been done on conservative access cavities instead of MOD cavities, as in the work of Nezir et al. (2024), it revealed that fiber-reinforced composites (EverXPosterior™) were significantly stronger than pure bulk-fill resin composite in endodontically treated mandibular molars [23] and can dramatically increase fracture toughness and flexural strength, sometimes comparable to those of indirect restorations [24]. In agreement with Hafez et al. (2025), our results supported the notion that EverX Flow + nanocomposite, at levels comparable to ceramic overlays and better than unrestored controls, allows micrometer-scale E-glass fibers to fracture-bridge [2]. Also, the mode of failure becomes more repairable, and peak loads can increase when SFRC (e.g., EverX Flow) is used as a core or when it is placed with optimized fiber orientation and adhesive protocols [25].

Based on the results of this research, teeth restored using the Stela resin-based composite (Group F1) exhibited intermediate fracture resistance, recorded at (561.75 ± 173.74 N). Notably, these values surpassed the fracture loads observed in the group restored with the traditional Z250 XT nanohybrid composite.

Stela’s material composition and curing mechanism affect our results, as unlike light-cured RCR, the self-cured polymerization mechanism of stela is a novel hydroperoxide-based initiator free of tertiary amine, allowing polymerization without regard for light penetration depth, in contrast to light-cured composites that require exogenous photon activation. Theoretically, compared with light-cured materials affected by light attenuation, this infinite depth of cure offers more thorough polymerization in deep cavities [24]. In addition, Stela’s documented benefits include a gap-free interface and long-term durability. The performance of Filtek Z250 XT in this study suggests limitations when used as a sole material for repairing the complex stress fields in endodontically treated teeth with large MOD cavities, despite its wear resistance and mechanical properties, due to its improved filler system containing surface-modified zirconia/silica particles and Nano silica particles [26].

The difference between three direct restoration groups is no statistically significant (*P* = 0.471–1.000), This variability reflects different composite curing polymerization kinetics, technique sensitivity of different placement technique of restorations, dentin substrate thickness and quality have effect on distribution of stresses and behavior of fracture, and sample size limitations, leading to variances between direct restoration groups was restricted [27].

Current guidelines indicate that adhesive resin composites may be a viable alternative for endodontically treated posterior restorations with a small-to-moderate amount of coronal destruction. It emphasizes not giving too much weight to differences observed among in vitro specimens during testing for fracture resistance [28].

Regarding the knowledge gathered from this study, it is essential to recognize a number of in vitro design limitations. The dynamic biological circumstances of the oral cavity, such as pH variations, salivary interactions, or the complex microbiota that contribute to material degradation over time, cannot be entirely replicated in a lab setting. Additionally, whereas static compressive loading offers a comparable baseline for fracture resistance, it ignores the oblique pressures and cyclic fatigue experienced during functional mastication. In order to maximize biomechanical performance, we recommended future studies should include thermomechanical fatiguing and a variety of preparation designs, such as the addition of a ferrule or varying pulp chamber depths. Additionally, to confirm the long-term viability and efficacy of various restorative materials in the oral environment, prospective longitudinal clinical trials are needed.

## Conclusions

Within the limitations of the current in vitro study, it was concluded that:

- Emax (CAD/CAM) Lithium Disilicate Endocrown showed significantly superior performance compared to all tested direct restorative materials, supporting their use in clinically demanding situations.
- Direct restoration materials demonstrated comparable performance, indicating their clinical interchangeability in routine restorative procedures.
- The fracture resistance of both short-fiber-reinforced composite (SFRC) EverX flow and a conventional nanohybrid Filtek Z250 XT resin-based composite (RBC) was better than that of the traditional nanohybrid Filtek Z250 XT alone.
- romising fracture resistance results of an innovative Stela self-adhesive bulk-fill resin-based composite (RBC).
- For further assessment of the biomechanical performance of the tested materials used in this study, it is recommended to perform thermomechanical fatigue and a variety of preparation designs.
- To confirm the long-term viability and efficacy of various restorative materials in the oral environment, prospective longitudinal clinical trials are needed.

## Data Availability

The datasets used and/or analyzed in this study are available from the corresponding author upon reasonable request.

## Abbreviations

ETT: Endodontically Treated Teeth
FR: Fracture Resistance
SFRC: Short-Fiber-Resin-Composite
RBC: Resin-Based Composite
MOD: Mesio-Occluso-Distal
CAD/CAM: Computer-Aided Design/Computer-Aided Manufacturing
YbF3: Ytterbium Trifluoride
Al2CaO: Calcium Aluminate
MEK: Methyl Ethyl Ketone
Al2CaO: Calcium Aluminate
MEK: Methyl Ethyl Ketone
Bis-GMA: Bisphenol A glycol dimethacrylate
UDMA: Urethane dimethacrylate
BIS-EMA: Ethoxylated bisphenol A glycol dimethacrylate
PEGDMA: Polyethylene glycol dimethacrylate
TEGDMA: Triethylene glycol dimethacrylate
